# Trajectories of antidepressant medication use in individuals before and after being granted disability pension due to common mental disorders- a nationwide register-based study

**DOI:** 10.1186/s12888-018-1628-8

**Published:** 2018-02-13

**Authors:** Syed Rahman, Michael Wiberg, Kristina Alexanderson, Jussi Jokinen, Antti Tanskanen, Ellenor Mittendorfer-Rutz

**Affiliations:** 1 Department of Clinical Neuroscience, Division of Insurance Medicine, Karolinska Institutet, SE-171 77 Stockholm, Sweden; 2 Department of Clinical Neuroscience, Division of Psychiatry, Karolinska Institutet, Karolinska University Hospital, Stockholm, Sweden; 3 Department of Clinical Sciences, Division of Psychiatry, Umeå University, Umeå, Sweden; 4 National Institute for Health and Welfare, Helsinki, Finland; 5 Department of Analysis and Forecast, Swedish Social Insurance Agency, Stockholm, Sweden

**Keywords:** Common mental disorders, Antidepressant, Epidemiology, Psychiatry in Europe, Disability pension, Sick-leave, Group-based trajectories

## Abstract

**Background:**

Early retirement caused by disability pension (DP) due to common mental disorders (CMDs) is frequent in European countries. Inadequate treatment, e.g., suboptimal antidepressant (AD) medication before DP can be crucial in such DP. This explorative study aimed to disentangle trajectories of AD based on defined daily dose (DDD) before and after granted DP, and to characterize the trajectories by socio-demographics and medical factors.

**Methods:**

All 4642 individuals in Sweden aged 19–64 with incident DP due to CMD in 2009–2010 were included. Trajectories of annual DDDs of AD were analysed over a 6-year period by a group-based trajectory method. Associations between socio-demographic or medical factors and different trajectories were estimated by chi^2^-test and multinomial logistic regression.

**Results:**

Five trajectories of ADs were identified. Three groups, comprising 34%, 34%, and 21% of the cohort, had constant AD levels before and after DP with mean annual DDDs of 29, 234, and 580, respectively. Two groups, each including 6% of the cohort, had increasing levels of DDDs, levelling off at around 1150 and 785 DDDs after DP. Particularly age, outpatient care due to mental diagnoses and DP diagnoses were significantly associated with different trajectories (*p* < 0.05). All the groups had a larger proportion of older individuals (> 50%, 45–64 years), except for the ‘increasing low’ group, where younger individuals were in majority (> 60%, 18–44 years), who more frequently exited labour market due to ‘anxiety disorders’, with lower education and more specialised healthcare before DP than the other groups.

**Conclusion:**

The heterogeneity among the five trajectory groups was partly explained by age, the severity of the mental disorder and the DP diagnoses. DDDs of ADs, though on different levels, varied marginally before and after granted DP in the majority. Moreover, AD levels were very low in one third of the individuals. Early identification and focus on the ‘increasing low’ group might be important in order to identify individuals at risk for further increase in annual DDDs of ADs even after granted DP, and might also contribute in prevention of DP. Further detailed research regarding different groups is warranted.

## Background

Common mental disorders (CMDs) constitute one of the most frequent reasons for disability pension (DP) claims and rank among the main medical causes of labour market marginalisation in OECD (Organisation for Economic Co-operation and Development) countries [1–6]. CMDs have a strong impact on individuals’ lives, e.g., adverse effect on social and occupational functioning [7], potentially leading to long-term sickness absence [8] and consequently even to disability pension [9].

CMDs are known to be positively affected by treatment and rehabilitation efforts and are likely to worsen with inactivity [10–12]. Adequate healthcare before DP, particularly adequate prescription of antidepressants (ADs) can reduce the burden of disease and hereby have an effect on improving work capacity and reducing premature exit from the labour market in terms of DP [13–15]. However, previous studies, which considered cognitive behavioural therapy (CBT), pharmacological treatment, and healthcare visits, have indicated inadequate treatment before being granted DP [16, 17].

Recent studies have reported an increase in psychotropic medications before DP due to mental disorders and an immediate decrease after DP [18–20]. It is likely that patterns of such medication may differ for different mental disorders and that such changes might be influenced by different socio-demographic and medical factors. To the best of our knowledge, no study has been conducted yet aiming at identifying potential variability of such trajectories before and after being granted DP and at focussing on one of the most frequent diagnostic DP groups, namely CMDs.

## Aim

The aims of this explorative study were to, among individuals granted DP due to CMD, 1) identify and describe different trajectories of AD over a 6-year period (3 years prior, and 3 years after being granted DP), and 2) analyse the heterogeneity, if any, between the trajectories by characterizing them with regard to socio-demographics and medical factors.

## Methods

### Study population

All individuals aged 19–64 and living in Sweden who were granted DP due to CMD for the first time during 2009–2010 (*N* = 4642) comprised the study base. CMD was defined as ‘depressive episode’ (F32), ‘recurrent depressive disorder’ (F33), ‘phobic anxiety disorder’ (F40), ‘other anxiety disorder’ (F41), ‘obsessive-compulsive disorder’ (F42), and ‘reaction to severe stress and adjustment disorder’ (F43) [21–24], based on the corresponding codes of the International Classification of Diseases (ICD) version 10 [25].

The study population was identified through register linkage at individual level using the unique ten-digit personal number of all residents in Sweden. Information was obtained from the following nationwide registers:

1) Longitudinal integration database for health insurance and labour market studies (LISA) (with information on: sex, age, education, country of birth, type of living area, family situation, and emigration) from Statistics Sweden.

2) (i) National patient register (date and main diagnosis of in- and specialized outpatient care); (ii) Drug register (information on prescribed dispensed drugs, including dates, dosages, amount, Anatomical Therapeutic Chemical (ATC) Classification System code [26], defined daily dose per dispensed pack; and (iii) Cause of death register (date of death) – all three from the National Board of Health and Welfare.

3) Micro-data for analyses of social insurance (MiDAS) (date and main disability pension diagnosis) from the National Social Insurance Agency.

### Antidepressants

Antidepressants were identified based on the respective ATC code (N06A) [26]. Levels of defined daily dose (DDD) of ADs per year during the 3 years before and 3 years after the date of being granted DP were assessed. The DDD, as defined by ‘the WHO collaborating centre for Drug Statistics Methodology’, is the tentative average maintenance daily dose for a drug, used as main indication in adults [27]. A time scale on an annual basis was introduced where t0 represented the first date of being on DP, t-1 to t-3 referred to the three respective years prior to DP, and t + 1 to t + 3 indicated the 3 years after. The total amount of DDDs for a given year was calculated by summing up the DDDs of any AD medication during that year. If more than one AD was purchased during a year, then annual DDDs of all AD were summed up together for that particular year.

### Drug purchase subsidies in Sweden

There is an upper limit of how much an individual has to pay out of pocket for medication during a 12-months period. The limit was between 100 and 126 Euros during the study period [28, 29]. Individuals can buy for up to a maximum of 3 months of prescribed medication at a time.

### Covariates

Socio-demographics (sex, age, education, country of birth, type of living area, and family situation) were measured on 31-Dec of the year preceding DP granting and categorised as shown in Table 1. Among the medical factors, previous healthcare was measured during the 3 years before starting DP (from t-3 to t0) and categorized as ‘yes’ and ‘no’. Regarding underlying diagnoses of previous healthcare, F00-F99 (ICD-10) codes were categorized as mental and all other as somatic diagnoses. The main DP diagnosis was the main one given when granting the DP (t0). We did not consider alteration in the main DP diagnosis, if any, during the study period. Diagnoses were categorized as indicated in Table 1. Annual in- and specialized outpatient healthcare use was considered as a proxy of the severity of the underlying disorder.

**Table 1:** Descriptive statistics for all women (*n* = 2897) and men (*n* = 1745) granted disability pension (DP) due to common mental disorders in 2009–2010 in Sweden (*N* = 4642)

*Characteristics, measured at the end of the year preceding granted DP*	All		Women		Men	
	*n* 4642	% 100	*n* 2897	% 62.4	*n* 1745	% 37.6
*Socio-demographic characteristics* ^*b*^						
Age^a^						
18–24	837	18.0	520	17.9	317	18.2
25–34	490	10.6	294	10.1	196	11.2
35–44	635	13.7	407	14.0	228	13.1
45–54	1112	24.0	730	25.2	382	21.9
55–64	1568	33.8	946	32.7	622	35.6
Education (years)^a^						
Compulsory (≤9)	1301	28.0	710	24.5	591	33.9
High school (10–12)	2134	46.0	1353	46.7	781	44.8
University (> 12)	1207	26.0	834	28.8	373	21.4
Country of birth^a^						
Sweden	3589	77.3	2314	79.9	1275	73.1
Other EU countries	278	6.0	180	6.2	98	5.6
Rest of the world	775	16.7	403	13.9	372	21.3
Type of living area^ac^						
Big cities	1852	39.9	1120	38.7	732	41.9
Medium sized cities	1576	34.0	988	34.1	588	33.7
Small towns/villages	1214	26.2	789	27.2	425	24.4
Family situation^a^						
Married^d^ living without children	743	16.0	496	17.1	247	14.2
Married^d^ living with children	967	20.8	598	20.6	369	21.1
Single^e^ living without children	2103	45.3	1188	41.0	915	52.4
Single^e^ living with children	478	10.3	420	14.5	58	3.3
Adolescents living with parents, < 20 years	351	7.6	195	6.7	156	8.9
*Medical characteristics*						
Main DP diagnosis^a^						
Depressive disorders	2150	46.3	1305	45.0	845	48.4
Anxiety disorders	1441	31.0	867	29.9	574	32.9
Stress-related mental disorders	1051	22.6	725	25.0	326	18.7
Previous healthcare^f^						
Mental inpatient care^a^	777	16.7	460	15.9	317	18.2
Specialized mental outpatient care^a^	2674	57.6	1588	54.8	1086	62.2
Somatic inpatient care	1349	29.1	864	29.8	485	27.8
Specialized somatic outpatient care^a^	3436	74.0	2225	76.8	1211	69.4

^a^Significant sex differences

^b^All socio-demographic variables are measured at t0

^c^Type of living area: big cities (Stockholm, Gothenburg and Malmö); medium sized cities (cities with more than 90,000 inhabitants within 30 km distance from the centre of the city); small cities/villages/rural

^d^Married includes all living with partner; cohabitant

^e^Single includes divorced, separated, or widowed

^f^Measured during t-3 to t0

### Disability pension in Sweden

All residents in Sweden aged 19–64 years, with a reduced work capacity due to disease or injury, can be granted DP from the Social Insurance Agency, for full- (100%) or part-time (75, 50, or 25% of ordinary working hours) [3]. People aged 19–29 years can be granted temporary DP if their work capacity is reduced due to disease or injury, not only for paid work but also to complete upper-secondary education, and individuals between 30 and 64 years of age can be granted permanent DP [3].

### Statistical analyses

We calculated the individual annual DDDs for each specific AD for the six studied years, considering the date of granted DP; t0. Then we summed up DDDs of all specific ADs the individual bought during a one-year interval. Annual cumulative DDDs exceeding 1500 (around 4 DDDs) were deemed unusual (might be due to special cases, large purchases before traveling abroad, or error in data). So any such annual DDDs were truncated at a level of 1500 (equals 194 yearly purchases).

Thereafter, we used group-based trajectory modelling to estimate trajectories of AD among individuals with incident DP due to CMD during 2009–2010, for six time points (i.e. within a six-year window, starting from 3 years before and ending at 3 years after the date being granted DP). These models estimate changes in AD patterns over time in multiple subgroups within the cohort and estimate a regression model for each discrete group, and assess proportions of individuals in each group.

Additionally, this flexible model allows for different polynomials of the outcome [30]. We used the Bayesian information criterion (BIC) to test the best-fitted model related to the number of groups between 2 to 8, and in parallel, we also considered the proportions of the individuals in each group. While six and seven group models had better BIC values compared to the five-group model, there were very few individuals in some of the groups. Therefore, the model with five groups was considered most appropriate.

**Fig. 1:**
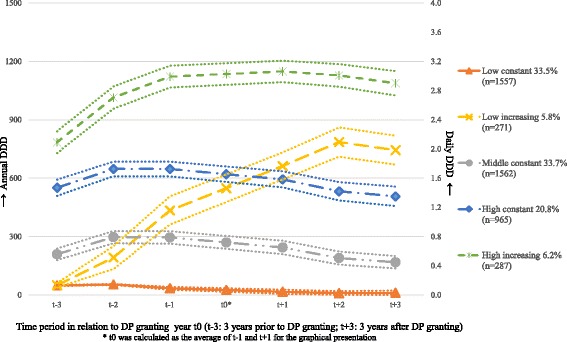
Trajectory groups of antidepressants according to annual defined daily doses (DDDs) and percentages of individuals with granted disability pension (DP) due to common mental disorders granted in 2009–2010 (*N* = 4642) within each trajectory group in Sweden. (The dotted lines represent 95% confidence intervals)

Probabilities for an individual to be assigned to a specific trajectory group were calculated. The highest estimated probability was used to decide each individual’s group belonging. Côté et al. recommend that the average probability for individuals of a trajectory group should be ≥0.70 [31]. Such average probability for individuals of our cohort was 0.89, indicating a very good fit. It should be noted that all the time points represented an interval of 1 year calculated on the basis of DP granting date (t0), but for the sake of graphical presentation, t0 was calculated as the average of t-1 and t + 1, though t0 represented a date and not an interval (Fig. 1).

We calculated descriptive statistics of the socio-demographics and medical factors prior to DP, after assessing potential sex differences in these factors by chi^2^-test. We then estimated their associations in each AD trajectory group by chi^2^-test and multinomial logistic regression. The likelihood ratio tests were used to evaluate whether socio-demographic and medical factors were associated with type of trajectory group in the full model. Moreover, Nagelkerke pseudo R^2^ values were estimated to evaluate the strength of these associations. By consecutively excluding and re-including each factor from the full model, we calculated differences in R^2^ for each factor in order to examine the contribution of a given factor to the full model.

Analyses were carried out for the entire study population with DP due to CMD regardless of grade (part-time/full-time) as sensitivity analyses revealed no differences in the trajectories between individuals with full-time and part-time DP.

In case of death or emigration during the study period, due to differences in the exposure time, the data on DDDs for an individual was considered as missing for the whole year of the event and onward.

Data processing was performed using statistical software SAS for Windows version 9.4 (SAS-based procedure “Traj” [32]) and SPSS for Windows version 22.0 (chi^2^-test and multinomial logistic regression).

## Results

In Table 1, descriptive statistics, including socio-demographics and medical factors for the cohort (individuals with incident DP due to CMD in 2009–2010) are presented. Two thirds of the cohort were women (62.4%) and 77.3% were born in Sweden. When granted DP, most of them were aged 45–64 years (57.8%), had proceeded to high school education (46.0%), lived in big cities (39.9%), and were single without any child at home (45.3%). A higher proportion of the women (14.5%) than of the men (3.3%) were single with children living at home. Depressive disorders were the most common (46.3%) DP diagnosis. A history of specialised outpatient care was more common than inpatient care due to mental or somatic diagnoses (57.6%, 74.0% compared to 16.7%, 29.1%, respectively).

Figure 1 shows the estimated five groups of different trajectories of AD. The groups were labelled as, ‘low constant’, ‘low increasing’, ‘middle constant’, ‘high constant’, and ‘high increasing’. Many of the individuals (‘low constant’ 33.5% of the cohort) had none or very low (less than 50) annual DDDs of AD. Nearly 6% percent (‘low increasing’) had very low annual DDDs 3 years before DP granting, and showed a steep increase in annual DDDs of AD up to 785 up until 2 years following DP. The group ‘middle constant’ (33.7%) had annual DDDs in between 200 and 300 during the study period. The group ‘high constant’ included 20.8% of the cohort, and had 500–600 DDDs per year throughout the study period. In the groups ‘middle constant’ and ‘high constant’, there was a slight decline in DDDs of AD following the DP. Six percent of the cohort had over 1000 DDDs per year since 2 years before DP granting until the end of follow-up (high increasing).

Table 2 describes the distribution of the socio-demographics and medical factors across the five identified trajectory groups. All socio-demographic and medical factors, but sex and previous healthcare due to somatic diagnoses, were significantly associated with different trajectory groups (*p* < 0.05) in the unadjusted analyses. In the full model, along with other variables, sex was significantly associated with the trajectory groups. The full model explained 17.2% of the variance between the groups (using Nagelkerke pseudo R^2^). The highest estimated difference of 5% was observed for ‘Previous mental outpatient care’ (diff. in R^2^ = 0.05), otherwise the individual factors, other than age and main DP diagnosis (diff. in R^2^ = 0.02), merely effected the full model independently.

**Table 2:** Distributions and associations of socio-demographic and medical characteristics in each trajectory group of antidepressants according to annual defined daily doses (DDDs), in individuals with disability pension (DP) due to common mental disorders granted in 2009–2010 (*N* = 4642) in Sweden

*Characteristics*	Low constant	Low increasing	Middle constant	High constant	High increasing	Pearson’s Chi-Square (*p*-value)^f^	Log-likelihood test Chi-Square (*p*-value)^g^	Diff. in R^2 *^
	*n* (%) 1557(33.54)	*n* (%) 271(5.84)	*n* (%) 1562(33.65)	*n* (%) 965(20.79)	*n* (%) 287(6.18)			
*Socio-demographic characteristics*								
Sex^a^								
Men	604 (38.8)	102 (37.6)	598 (38.3)	347 (36.0)	94 (32.8)	5.2 (0.26)	10.7 (0.03)	0.002
Women	953 (61.2)	169 (62.4)	964 (61.7)	618 (64.0)	193 (67.2)			
Age^a^								
18–24	286 (18.4)	112 (41.3)	305 (19.5)	101 (10.5)	33 (11.5)	207.4 (< 0.001)	109.1 (< 0.001)	0.021
25–34	135 (8.7)	44 (16.2)	172 (11.0)	104 (10.8)	35 (12.2)			
35–44	202 (13.0)	23 (8.5)	207 (13.3)	144 (14.9)	59 (20.6)			
45–54	348 (22.4)	42 (15.5)	360 (23.0)	277 (28.7)	85 (29.6)			
55–64	586 (37.6)	50 (18.5)	518 (33.2)	339 (35.1)	75 (26.1)			
Education (years)^a^								
Compulsory (≤9)	495 (31.8)	114 (42.1)	444 (28.4)	198 (20.5)	50 (17.4)	88.3 (< 0.001)	29.7 (< 0.001)	0.006
High school (10–12)	649 (41.7)	110 (40.6)	738 (47.2)	485 (50.3)	152 (53.0)			
University (> 12)	413 (26.5)	47 (17.3)	380 (24.3)	282 (29.2)	85 (29.6)			
Country of birth^a^								
Sweden	1171 (75.2)	219 (80.8)	1162 (74.4)	792 (82.1)	245 (85.4)	40.8 (< 0.001)	57.3 (< 0.001)	0.011
Other EU25 countries	106 (6.8)	12 (4.4)	95 (6.1)	55 (5.7)	10 (3.5)			
Rest of the world	280 (18.0)	40 (14.8)	305 (19.5)	118 (12.2)	32 (11.1)			
Type of living area^a,b^								
Big cities	683 (43.9)	97 (35.8)	586 (37.5)	375 (38.9)	111 (38.7)	21.9 (< 0.01)	20.6 (< 0.01)	0.004
Medium sized cities	485 (31.1)	95 (35.1)	553 (35.4)	353 (36.6)	90 (31.4)			
Small towns/villages	389 (25.0)	79 (29.2)	423 (27.1)	237 (24.6)	86 (30.0)			
Family situation^a^								
Married^c^ living without children	250 (16.1)	32 (11.8)	248 (15.9)	168 (17.4)	45 (15.7)	91.9 (< 0.001)	27.1 (0.04)	0.005
Married^c^ living with children	311 (20.0)	30 (11.1)	324 (20.7)	233 (24.1)	69 (24.0)			
Single^d^ living without children	697 (44.8)	136 (50.2)	718 (46.0)	415 (43.0)	137 (47.7)			
Single^d^ living with children	164 (10.5)	23 (8.5)	160 (10.2)	106 (11.0)	25 (8.7)			
Adolescents living with parents, < 20 years	135 (8.7)	50 (18.5)	112 (7.2)	43 (4.5)	11 (3.8)			
*Medical characteristics*								
Main DP diagnosis^a^								
Depressive disorders	630 (40.5)	117 (43.2)	727 (46.5)	521 (54.0)	155 (54.0)	140.7 (< 0.001)	75.5 (< 0.001)	0.015
Anxiety disorders	436 (28.0)	117 (43.2)	507 (32.5)	290 (30.1)	91 (31.7)			
Stress-related mental disorders	491 (31.5)	37 (13.7)	328 (21.0)	154 (16.0)	41 (14.3)			
Previous mental inpatient care^e^								
Yes	142 (9.1)	63 (23.2)	274 (17.5)	229 (23.7)	69 (24.0)	118.6 (< 0.001)	31.5 (< 0.001)	0.006
No	1415 (90.9)	208 (76.8)	1288 (82.5)	736 (76.3)	218 (76.0)			
Previous mental specialized outpatient care^e^								
Yes	630 (40.5)	183 (67.5)	942 (60.3)	691 (71.6)	228 (79.4)	336.5 (< 0.001)	244.7 (< 0.001)	0.048
No	927 (59.5)	88 (32.5)	620 (39.7)	274 (28.4)	59 (20.6)			
Previous somatic inpatient care^e^								
Yes	416 (26.7)	90 (33.2)	458 (29.3)	299 (31.0)	86 (30.0)	8.3 (0.08)	4.5 (0.4)	0.001
No	1141 (73.3)	181 (66.8)	1104 (70.7)	666 (69.0)	201 (70.0)			
Previous somatic specialized outpatient care^e^								
Yes	1153 (74.1)	203 (74.5)	1144 (73.2)	723 (74.9)	213 (74.2)	1.02 (0.91)	1.7 (0.8)	0.000
No	404 (25.9)	68 (25.1)	418 (26.8)	242 (25.1)	74 (25.8)			

^*^Difference in Nagelkerke pseudo R^2^ between model including tested variable and model without tested variable. Nagelkerke pseudo R^2^ for full model is 0.172

^a^Measured at t0

^b^Type of living area: big cities: Stockholm, Gothenburg and Malmö; medium sized cities: cities with more than 90,000 inhabitants within 30 km distance from the centre of the city; small cities/villages

^c^Married includes all living with partner; cohabitant

^d^Single includes divorced, separated, or widowed

^e^Measured during t-3 to t0

^f^Crude model

^g^Mutually adjusted model

All the groups had a larger proportion of older individuals (> 50%, 45–64 years), except for the ‘low increasing', where younger individuals constituted the absolute majority (>60%, 18-44 years), and notably 41.3% of those in this group were in the 18-24 age range. This group also had fewer individuals (17.3%) who have been to university compared to the other groups, whereas the ‘high increasing’ group had the highest proportions of individuals having attended high school or university (53.0% and 29.6%, respectively). In the ‘low increasing’ group, 80% of the individuals were single and did not have any children. Other socio-demographic factors were fairly equally distributed among all five groups.

Regarding the main DP diagnosis, the ‘low increasing’ group had equal proportions of depressive and anxiety disorders (43.2% each), whereas in all other trajectory groups, depressive disorders dominated. The percentage of the individuals with ‘stress-related mental disorders’ as main DP diagnosis was largest in the ‘low constant’ (31.5%) and followed by the ‘middle constant’ (21.0%) groups. The proportions of the individuals from the ‘high increasing’ group who had had previous in- or specialized outpatient care due to mental diagnoses were approximately twice as high compared to the proportions of the ‘low constant’ group (24.0%, 79.4% and 9.1%, 40.5%, respectively).

Approximately 41% (*n* = 1906) of the study population did not receive in- or specialized psychiatric care during the 3 years prior to DP grant. Further analyses showed that this group consisted individuals somewhat older (mean age 49 vs 44 years), with better educational level (university education: 32% vs 26%) than the whole study population (data not shown). Regarding the main DP diagnosis, more individuals without previous psychiatric specialised health care were granted DP due to stress-related mental disorders than in the whole study population (31.8% vs 22.6%), and fewer received DP due to anxiety disorders (22.5% vs 31%) (data not shown). Trajectory analyses revealed this group had similar patterns of trajectory groups, but larger proportions of ‘low constant’ and ‘high constant’ group (38.5% vs 33.5%, and 24.3% vs 20.8%, respectively) and smaller proportions of ‘middle constant’ trajectory groups (24.1% vs 33.7%), and received lower levels of ADs. Multinomial regression suggested similar associations of covariates with trajectory groups as in the whole study population (data not shown).

## Discussion

### Main findings

In this explorative study, we identified five different trajectories of DDDs of ADs over a 6-year period among all 4642 individuals granted DP due to CMDs during 2009–2010. For the vast majority of individuals (89%), DDDs of ADs – though on different levels – varied only slightly before and after granting of a DP. Out of them about a third of the individuals, who more often had stress-related DP diagnoses and less psychiatric care, received very low levels or no ADs during the years around the time of DP grant. Two smaller groups (6% each) registersshowed increases of DDDs up to granting of the DP, in one group DDDs levelled off afterwards and in the other group they kept increasing. Individuals in this latter group tended to be younger and were more likely to have an anxiety disorder as a DP diagnosis.

### Methodological considerations

To the best of our knowledge, this is the first study attempting to disentangle different groups of trajectories related to the amount of ADs during the years before and after being granted DP due to CMD. Previous studies have not discriminated between specific DP mental diagnoses and have used other statistical methods, which are not capable to identify groups with varying trajectories [18, 19].

Main strengths of this study include the use of high quality population-based nationwide registers [33–37] with longitudinal data linked at individual level. Further strengths result from the use of a large study group which comprised all individuals aged 18–64 from whole Sweden who were granted DP due to CMD during the studied exposure years (2009–2010). This means the study is not merely based on a sample. The register data also means that the study was not affected by recall bias regarding exposure and outcome measures. Moreover, there was no loss to follow up and DP diagnoses were set by the treating and certifying physician through thorough assessments of the patients’ disease, functioning, and work incapacity. An additional advantage is that a wide range of socio-demographic and medical factors could be included as potential confounders. Another important strength of our study is that a potential social gradient for AD purchase can be considered negligible. First, drug purchase is considerably subsidised in Sweden and the level of these subsidies did not change much during study period. Moreover, analyses were controlled for socio-economic factors.

Regarding limitations, the validity of sick-leave and DP diagnoses is often discussed; however, few related studies are carried out so far. A Swedish study from 1991 concluded that sick-leave diagnoses have high validity when compared to the diagnoses from medical records [38]. Additionally, granting of DP is preceded by a long process of medical evaluation and work-capacity assessments [3]. Moreover, due to the stigma around mental diagnoses [39, 40], we assume that a mental diagnosis is given as a main DP diagnosis only when the patient actually has a mental disorder and when the main reason of work disability cannot be attributed to a somatic diagnosis [41]. On the other hand, this implies that some people with CMD might not have been given CMD as the main DP diagnosis on the DP certificate. Thus, they would not be included in this study. This can also be seen as a strength, as our cohort of individuals with DP due to CMD is more strictly defined. Another limitation is that, as in all studies using drug registers, we have no information on whether the individual actually used the dispensed AD. However, as the AD cannot be prescribed for more than 3 months at a time, it is plausible that the patient would not repeatedly have bought medication not used.

A point to be noted is, that we did not have the possibility to measure formally the severity of CMD, we have rather used information on in- or specialized outpatient healthcare use as a proxy of severity. Concerning the possibility of returning to work after DP granting, it should be noted that DP is a permanent measure for individuals aging from 30 to 64 years (around 75% of the study population). Moreover, as we have considered only the individuals who were on DP during the whole follow-up, it is unlikely for even the younger individuals (with temporal DP) to have returned to work. However, we do not have the information if some have returned to work after the follow-up period. Also, looking at the role of employers would have given further insight, but unfortunately, we do not have data on different aspects of employers.

### Discussions of findings

The study revealed heterogeneity between the five groups identified based on the amounts of ADs. A third of the individuals in our study population had either no or very low annual DDDs of ADs during the 6 years of observation. This finding is surprising and may suggest that there is a possibility that these individuals might not have received optimal pharmacological treatment by ADs before being granted DP, which is in line with previous reports [16]. On the other hand, the individuals, if treated without pharmacotherapy, e.g., psychotherapy only, or other occupational therapy during some years of observation and later with ADs, might show low DDDs during the study period. Moreover, some in the cohort were prescribed other psychotropic drugs than ADs. Around 8–11% (depending upon the trajectory group) of the individuals in this cohort were prescribed anxiolytics or sedatives, separately or along with AD (data not shown). There is also a possibility of poor compliance to ADs due to their potential side effects, e.g., weight gain, decreased libido, diarrhoea, agitation etc. [42–44], which might have led to low annual DDDs. Finally, one should also consider that individuals might have improved clinically through AD treatment, but not regarding their work capacity [45–47].

Our analyses showed that most trajectory groups had relatively stable annual DDDs of AD around the time when granted DP. This is in contrast to findings from a Finnish project, where use of AD substantially increased before and decreased immediately after granted DP, especially among those with a mental DP diagnosis [18, 19]. These discrepancies in findings might arise from the differences in DP diagnoses, i.e. in our study the focus was on CMDs, while other previous studies included all mental disorders. One group differed considerably from the patterns of the other trajectory groups, i.e., the ‘low increasing’ group which showed a steep increase in DDDs of AD since the beginning of the study period (t-3) up to DP granting and continued to increase after that.

The study findings also indicate heterogeneity regarding socio-demographics and medical factors between the trajectory groups. Among the socio-demographic variables, age had the strongest association with trajectory groups in the full model. There were also observable differences in educational level and family situation between the trajectory groups. Differences in educational level or in the family status in the ‘low increasing’ trajectory group might have been due to the younger age distribution in that group. Regarding the level of education, individuals with higher educational level are likely to have more employment security regarding a current job, or even a greater choice of alternative jobs if they are not able to remain at the present one due to health reasons [48]. It is further not unlikely that these individuals may remain longer time at work with a later pensionary age despite a higher clinical severity and higher AD doses than their low educated peers.

Depressive disorders were the most common DP diagnosis in all the trajectory groups. However, anxiety disorders contributed equally much in the ‘low increasing’ group. This may be because many of them were young, and anxiety disorders usually have an earlier onset and they are detected earlier in life [49–51]. Previous mental inpatient or specialized outpatient healthcare was proportionate to the amount of AD, i.e., the highest AD purchases were found in the constant ‘high group’ and the lowest in the ‘low constant’ group. It might be necessary to pay special attention to the ‘low increasing’ group because they also had a high use of previous mental healthcare. As this includes the youngest among all trajectory groups, we expected that they would less frequently be using healthcare [52, 53]. Thus, our results suggest that individuals belonging to this group might have suffered from severe mental disorders several years before granted DP, and that the disorders worsened with time leading to DP. Notably, this group also had the highest level of previous somatic inpatient care. Such high use of previous healthcare suggests that further research should also focus on somatic comorbidity. On the other hand, 20–60% of the individuals, depending on the trajectory groups, did not receive any specialized mental healthcare during 3 years prior to DP. This is not in accordance with the Swedish sick-leave guidelines [54], stating that all patients on sickness absence due to depression for more than 6 months, should be referred to a specialist. This may indicate that these individuals might have not received optimal treatment during the pre-DP years, leading to further worsening of work capacity and later resulting in exclusion from the labour market by being granted DP [16].

A large proportion of the study population (41%), who were more often granted DP due to stress-related mental disorders, did not receive psychiatric in- or specialized outpatient care during the 3 years before being granted DP. This is consistent with previous research suggesting that individuals with work disability due to stress-related mental disorders have a lower proportion of specialized health care than individuals with other common mental disorders e.g., depressive disorders [55].

## Conclusion

The study identified five different groups according to the annual DDDs of ADs among individuals granted DP due to CMD. The five groups were heterogeneous regarding socio-demographics and medical factors, particularly regarding age, the severity of the mental disorder and the DP diagnoses. In the majority of the individuals within a trajectory group, the levels of DDDs of ADs varied only marginally before and after being granted DP. The study also posts a query regarding optimal pharmacological treatment for CMD during the pre-DP years, which in turn may contribute in prevention of premature exit from the labour market due to common mental disorders.

## Abbreviations

AD: Antidepressant
ATC: Anatomical therapeutic chemical
BIC: Bayesian information criterion
CMD: Common mental disorder
DDD: Defined daily dose
DP: Disability pension
ICD: International classification of diseases
LISA: Longitudinal integration database for health insurance and labour market studies
MiDAS: Micro-data for analyses of social insurance
OECD: Organisation for Economic Co-operation and Development
